# Phase preservation of orbital angular momentum of light in multiple scattering environment

**DOI:** 10.1038/s41377-024-01562-7

**Published:** 2024-08-26

**Authors:** Igor Meglinski, Ivan Lopushenko, Anton Sdobnov, Alexander Bykov

**Affiliations:** 1 College of Engineering and Physical Sciences, Aston University, Birmingham, B4 7ET UK; 2 Optoelectronics and Measurement Techniques, University of Oulu, Oulu, FI-90014 Finland

**Keywords:** Optical techniques, Optical physics

## Abstract

Recent advancements in wavefront shaping techniques have facilitated the study of complex structured light’s propagation with orbital angular momentum (OAM) within various media. The introduction of spiral phase modulation to the Laguerre–Gaussian (LG) beam during its paraxial propagation is facilitated by the negative gradient of the medium’s refractive index change over time, leading to a notable increase in the rate of phase twist, effectively observed as phase retardation of the OAM. This approach attains remarkable sensitivity to even the slightest variations in the medium’s refractive index (*∼*10^*−*6^). The phase memory of OAM is revealed as the ability of twisted light to preserve the initial helical phase even propagating through the turbid tissue-like multiple scattering medium. The results confirm fascinating opportunities for exploiting OAM light in biomedical applications, e.g. such as non-invasive trans-cutaneous glucose diagnosis and optical communication through biological tissues and other optically dense media.

## Abstract (web summary)

Structured light carrying OAM exhibits high sensitivity to refractive index variations and phase memory in scattering media, with strong potential for non-invasive glucose diagnosis and optical communication.

## Graphical abstract

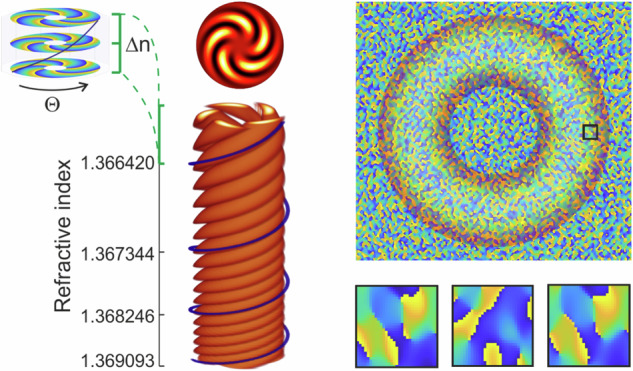

## Introduction

While using polarized light for the studies of different properties of matter in astrophysics, material science, and biomedicine has already a long history^1^, the shaped light possessing orbital angular momentum (OAM)^2^ has been added to the potential practical toolkit only recently^3^. The shaped OAM light plays an emerging role in both classical and quantum science, and offers fascinating opportunities for exploring new fundamental ideas, as well as for being used in practical applications^4^. The research in the field of shaped light with OAM achieved high recognition in the generation and characterization of the exotic vector laser beams^5^, improving telecommunication technologies^6^, and optical trapping of cells^7^ and micro-particles^8^. Despite the technical challenges, the shaped OAM light is emerged as one of the most exciting front lines of contemporary research, promising to defy the deficiencies of current optical sensing techniques^9^. OAM-based twisted light holds great potential for various biomedical applications in the field of biological tissue diagnosis. Apart from the precise optical manipulation and sorting of biological particles and cells utilizing optical optical tweezers^10,11^ the emerging biomedical applications based on OAM-based twisted light include imaging and microscopy^12^, generation twisted light from typical on-chip devices^13^, optical communication in biological media^14^ and other^9,15^.

Recent studies have been devoted to investigating the influence of OAM light, including light with Pancharatnam-Berry phase^16,17^, on tissue transmittance in both ballistic and diffusive regions^18–20^. Notably, it was demonstrated that transmittance increases in relation to the topological charge of OAM light^21–23^. This effect can vary depending on the scattering properties of different tissues^24^. Additionally, it was revealed that light with a topological charge can affect Raman spectra^25^.

In light of the pivotal contributions to the field of OAM and its interactions with scattering media, the current study integrates and builds upon foundational research that explores the effects of scattering (*µ*_s_) and absorption (*µ*_a_) coefficients, and the transmission characteristics of OAM light through scattering particles and biological tissues. Acknowledging the diversity of OAM light’s behavior in response to different topological charges (*ℓ*) and scattering regimes—ranging from ballistic to snake-like and diffusive—this study aims to provide further insights into the intricate dynamics of OAM light’s phase preservation within complex media. We extend our gratitude to the mentioned pioneering works that have laid the groundwork for this exploration, ensuring a comprehensive understanding of the nuanced impact of scattering and absorption on the transmission efficiency of OAM light in such environments.

More specifically, the current study aims to clarify the novel utilization of OAM for probing purposes, emphasizing its distinct advantages over conventional phase patterns and establishing a new paradigm in optical probing of complex media. Through our rigorous exploration and detailed comparative analysis, we underscore the profound implications of employing OAM light for probing applications, marking a significant contribution to the field of optical sciences.

In delineating the applications of OAM light, our research distinctly emphasizes the dual utility of OAM in both probing complex media and enhancing optical communications. While the capacity of OAM light to carry information through its topological charge has been extensively harnessed in the field of optical communications^4,26,27^, offering a pathway to increase bandwidth and data transmission efficiency, its application in probing represents a novel and equally transformative avenue. Unlike its use in communications, where the focus is on the transmission of data over distances with minimal loss or interference, the probing application of OAM light leverages its unique phase structure and topological stability to investigate and elucidate the intrinsic properties of materials, particularly through scattering or turbid environments.

The key distinction lies in the objective: in probing, the topological features of OAM light facilitate the detection of minute changes within a medium, enabling advances in non-invasive diagnostics and material characterization. This contrasts with communications, where the emphasis is on preserving the integrity of transmitted information. However, the underlying principle that makes OAM light advantageous in both domains is its resilience to phase distortion and its capability to maintain topological charge over propagation, showcasing the versatility of OAM light in addressing diverse scientific and technological challenges.

Through our exploration, we aim to highlight this distinction, showcasing the probing capabilities of OAM light as a separate and significant application area. This distinction underscores the breadth of OAM light’s applications beyond communications, paving the way for innovative approaches to optical probing that exploit the unique properties of OAM for scientific discovery and technological advancement.

We explore the prediction capacities of OAM of Laguerre–Gaussian (LG) beams by analyzing their helical wavefront change along propagation through a tissue-like medium. In the frame of the paraxial approximation LG beam is defined as^28–30^:

1 $$\begin{array}{c}L{G}_{p}^{{\ell}}\left(\rho ,\phi ,z\right)=\sqrt{\frac{2p!}{\pi \left(\left|{\ell}\right|+p\right)!\,{w}^{2}\left(z\right)}}{\left[\frac{\rho \sqrt{2}}{w\left(z\right)}\right]}^{\left|{\ell}\right|}{L}_{p}^{\left|{\ell}\right|}\left[\frac{2{\rho }^{2}}{{w}^{2}\left(z\right)}\right]\exp \left[-\frac{{\rho }^{2}}{{w}^{2}\left(z\right)}\right]\times \\ \times \exp \left[i\left(2p+\left|{\ell}\right|+1\right)\arctan \left(z/{z}_{R}\right)\right]\exp \left[\frac{-{ik}{\rho }^{2}z}{2\left({z}^{2}+{z}_{R}^{2}\right)}\right]\exp \left[-i{\ell}\phi \right]\exp \left[-{ikz}\right]\end{array}$$

Here, $k=2\pi /\lambda$, $\lambda$ is the wavelength of laser radiation, $w\left(z\right)=w\left(0\right)\sqrt{1+{\left(z/{z}_{R}\right)}^{2}}$, ${z}_{R}=\pi {w}^{2}\left(0\right)/\lambda$, $w\left(0\right)$ corresponds to the zero-order Gaussian beam waist and is adjusted to fit the experimental image, {*ρ,ϕ,z*} represents the cylindrical coordinates system utilized for characterization of beam propagation along *z*-axis in terms of radial (*ρ*) and angular (*ϕ*) coordinates.

Phase evolution of the LG beam along its propagation in a medium is defined as^30^:

2 $$\Psi \left(\rho ,\phi ,z\right)={\rm{arg}}\left(L{G}_{p}^{{\ell}}\left(\rho ,\phi ,z\right)\right)=\frac{-k{\rho }^{2}z}{2\left({z}^{2}+{z}_{R}^{2}\right)}{{-}}{\ell}\phi -{kz}+G\left(z\right)$$

where $G\left(z\right)=\left(2p\,{\mathscr{+}}\,{\ell}{\mathscr{+}}\,1\right)\arctan \left(z/{z}_{R}\right)$ corresponds to the Gouy phase^31^. The helical phase of an LG beam refers to the phase front that winds around the beam’s axis, is a result of the azimuthal phase term in the beam’s electric field expression, which corresponds to the topological charge (*ℓ*) and radial index (*p*).

The predictive capacity of OAM light—its ability to foretell changes within a medium based on alterations in its phase structure—positions it as a powerful tool for non-invasive diagnostics^32^. This capability is of paramount importance in biomedical applications, where the early detection and characterization of disease at the molecular level can significantly impact patient outcomes^33^.

Furthermore, current study aims to clarify the dual utility of OAM light, distinguishing between its use in probing for biomedical information revelation and its well-established role in communications. By exploring and emphasizing these distinct applications, we highlight the versatility of OAM light and its potential to open new avenues in scientific research and technological development.

## Results

**Fig. 1:**
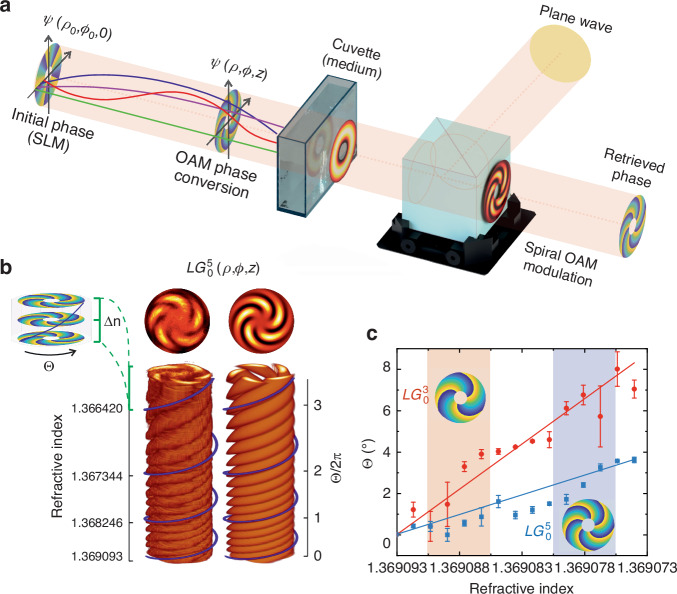
Twist of OAM induced by gradual change of the medium refractive index. **a** Schematic representation of the central part of the Mach–Zehnder interferometer experiment: the LG beam, carrying OAM imparted by a spatial light modulator (SLM), traverses a cuvette containing the probed medium; interference between the transmitted LG beam and a reference plane wave (an initially expanded Gaussian beam) is then analyzed for intensity and/or retrieved phase distributions. **b** Spiral modulation of the intensity of $L{G}_{0}^{5}$ beam observed experimentally (left) during the gradual increase of the medium’s refractive index, alongside the prediction from theoretical modeling^35,36^ (right); blue line corresponds to an increased rate of twist of OAM light along LG beam propagation in the medium. The inset (left) depicts the relative phase twist ($\varTheta =\frac{\varPsi }{{\ell}}$) of the OAM of $L{G}_{0}^{5}$ beam during gradual increase of the medium refractive index ($\Delta n=3.69\times {10}^{-4}$). **c** Relative phase twist of OAM of $L{G}_{0}^{3}$ (circles) and $L{G}_{0}^{5}$ (squares) beams during the gradual increase of medium refractive index within a range of $2\times {10}^{-5}$; Highlighted colored areas $\Delta n=5\times {10}^{-6}$) feature corresponding twist of OAM for $L{G}_{0}^{3}$ and $L{G}_{0}^{5}$ beams, respectively

The LG beam retains its helical structure (Eq. 2) while propagating through the transparent medium, preserving the phase composition Ψ(*ρ,ϕ,z*) associated with OAM and the characteristic helix-like wavefront (Fig. 1). Here and below we consider, so-called, scalar LG beams^29^ with homogeneous annular polarization distribution ($$L{G}_{0}^{3}$$ and $$L{G}_{0}^{5})$$.

The observed OAM phase retardation along with a notable increase in the rate of relative twist of phase ($\varTheta =\frac{\varPsi }{{\ell}}$) of the LG beam (see Fig. 1b), as it propagates through the medium with the negative gradient of the temporal change of the refractive index, are due to spatial dispersion of photon trajectories, defined as^30,34^:

3 $$\begin{array}{c}L\left({r}_{0},{\varphi }_{0},{\zeta }_{s}\right)={\int }_{0}^{{\zeta }_{s}}\sqrt{{\left(\frac{{dr}}{d\zeta }\right)}^{2}+{r}^{2}{\left(\frac{d\varphi }{d\zeta }\right)}^{2}}d\zeta \\ r\left(\zeta \right)={r}_{0}\sqrt{1+4{\zeta }^{2}},\varphi \left(\zeta \right)=\frac{{\ell}}{2{r}_{0}^{2}}\arctan \left(2\zeta \right)+{\varphi }_{0}\end{array}$$

Here, $$L\left({r}_{0},{\varphi }_{0},{\zeta }_{s}\right)$$ is the length of spiral-like trajectory along LG beam propagation; *r*_0_*,φ*_0_ define the radial and angular coordinates of the trajectory at the starting point ($$z=0)$$; $$r=\frac{\rho }{w\left(0\right)}$$ and $$\zeta =\frac{z}{k{w}^{2}\left(0\right)}$$ are the dimensionless cylindrical coordinates; ζ_*s*_ defines the point to which trajectory length is evaluated; $$r\left(\zeta \right)$$, $$\varphi \left(\zeta \right)$$ are the corresponding coordinates at the trajectory ($$0\le \zeta \le {\zeta }_{s}$$), the direction of which ($${\bf{p}}=\{{p}_{\rho },{p}_{\phi },{p}_{z}\}$$) defined as^28,30^:

4 $$\begin{array}{c}{p}_{\rho }=\frac{\omega k\rho z}{\left({z}_{R}^{2}+{z}^{2}\right)}{\rm{|}}L{G}_{p}^{{\ell}}\left(\rho ,\phi ,z\right){{\rm{|}}}^{2}\\ {p}_{\phi }=\frac{\omega l}{\rho }{\rm{|}}L{G}_{p}^{{\ell}}\left(\rho ,\phi ,z\right){{\rm{|}}}^{2}\\ {p}_{z}=\omega k{\rm{|}}L{G}_{p}^{{\ell}}\left(\rho ,\phi ,z\right){{\rm{|}}}^{2}\end{array}$$

where *ω* is the angular frequency of the light.

In the LG beam, the photon trajectories follow the spiral paths around the optical axis of the beam^30,34^, as presented in Fig. 1a. Gradual increase of the medium’s refractive index gives a proportional elongation of these spiral-like trajectories and increased length deviation between them. The increment of the phase component $\left(\frac{-k{\rho }^{2}z}{2\left({z}^{2}+{z}_{R}^{2}\right)}-{kz}\right)$ along the longer trajectories leads to the emergence of a gradient in the LG beam phase (Eq. 2) that in turn manifests itself as a twisting effect in its transverse distribution (see Fig. 1b). Accordingly, when the characteristic size of the medium (e.g., the thickness of the medium) is much larger than the wavelength ($d\gg \lambda$) even a minor variation in the medium refractive index leads to a significant alteration of the transverse spatial phase distribution of the LG beam (see Fig. 1c). A prominent twist of OAM is observed experimentally up to minuscule changes of the medium refractive index (Δ*n* = 10^−6^ (see Fig. 1c)). A gradual decrease in the medium’s refractive index induces a spiral modulation of the LG beam, observed via its interference with a plane wave (an expanded Gaussian beam), as an increased rate of OAM twist (see Fig. 1b). The experimental results obtained align closely with the theoretical predictions^35–37^as presented in Fig. 1b. In the case of the LG beams with lower topological charge (*ℓ* = 3), the Poynting vector trajectories tend to be less tightly wound around the optical axis^30,34^. Therefore, the OAM twist for $L{G}_{0}^{3}$ beams with lower topological charge exhibits higher accuracy in predicting even negligible changes in the refractive index compared to the $L{G}_{0}^{5}$ beam (see Fig. 1c).

The transition of the beam from a constant phase gradient to a spiral shape, introduced as ‘OAM phase conversion’, illustrates the imparting of OAM to the light beam. This transformation is facilitated by introducing a helical phase structure, resulting in a characteristic spiral wavefront. This process is achieved through phase modulation using optical elements such as a spatial light modulator (SLM), which encodes the beam with angular momentum. The spiral pattern, therefore, is not merely a product of focusing or defocusing effects but a deliberate modification to endow the light beam with OAM.

The ability of the LG beam to maintain this spiral phase structure through various media highlights its potential for sensitive detection applications, from non-invasive diagnostics to communication through scattering materials.

In this experimental study, we demonstrate the effects of a gradual decrease in the medium’s refractive index on the propagation of an LG beam, observed through the accelerated OAM twist. This phenomenon is meticulously replicated in our experiments by employing a temperature-dependent ethanol–water solution, showcasing the sensitivity of OAM light to even minor refractive index variations. Such controlled manipulation of the refractive index serves as a robust model for simulating physiological changes in biological tissues, where similar refractive index variations can occur due to biochemical alterations, including glucose concentration shifts in interstitial fluids.

The relevance of this approach extends beyond the confines of our experimental setup, offering a window into the potential biomedical applications of OAM light. Specifically, it underscores the capacity of OAM-based probing techniques to detect subtle changes in tissue properties, highlighting the feasibility of employing this method for non-invasive diagnostics. This principle is akin to the use of optical polarimetry for non-invasive glucose monitoring in the anterior chamber of the eye^38,39^.

While the twist of OAM of LG beam along propagation through a transparent medium (see Fig. 1) primarily arises due to the gradual increase of the medium refractive index, causing a corresponding enhancement of the lengths of spiral-like photon trajectories (Eq. 3), in a turbid medium, the scattering of the LG beam leads to the speckle pattern formation. Arising from the superposition of partial components of the helical wavefront, the speckle interference pattern manifests spatially varying intensity and phase distributions. This occurrence contributes to the disruption of the LG beam helical structure composition in low-scattering media and its complete degradation in multiple-scattering media (as presented in Fig. S1 and see Supplementary Note 1).

Disperse turbid media exhibiting low or multiple scattering of light are characterized by their optical depth (∼*d*/*l*^∗^), which serves as a quantitative measure of the extent of attenuation and scattering strength experienced by light along its propagation through. Here, *d* is the thickness of the scattering medium and *l*∗ is the transport mean free path. A commonly used guideline is that if *d*/*l*∗ ∼ 10 or larger the medium is considered as multiple or diffuse scattering^40^, whereas for the single and an intermediate (‘snake-like photons’) scattering *d*/*l*∗ is lower (∼3–6).

**Fig. 2:**
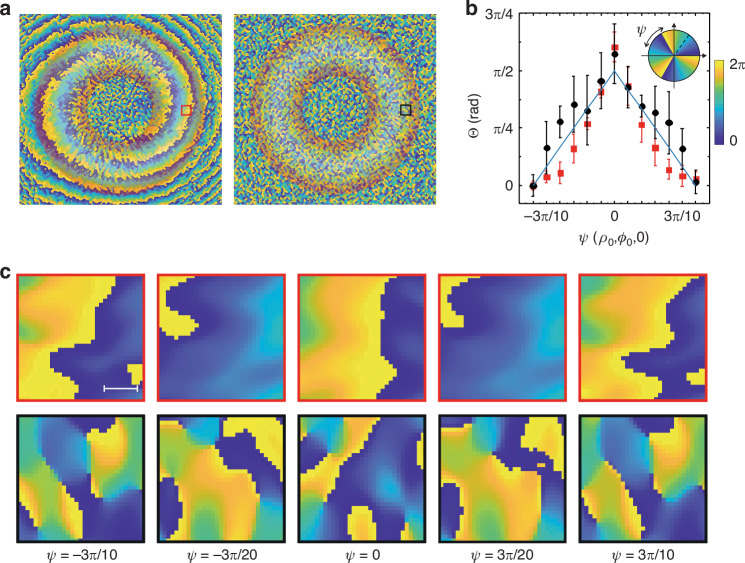
Phase memory of OAM of light in multiple scattering. **a** Phase distribution (speckle patterns) observed experimentally for the $L{G}_{0}^{3}$ beam propagated through the low (*d*/*l*∗ = 2) scattering (left) and multiple (*d*/*l*∗ = 9.6) scattering (right) media. The axial annular zone (embossed by contours) corresponds to the $L{G}_{0}^{3}$ beam as if it were passing through a medium devoid of scattering. **b** Phase variations manifest at the single speckle grain within a deliberately chosen sector of the $L{G}_{0}^{3}$ axial annular area for low scattering (indicated by black circles) and multiple-scattering (represented by red squares) media. The observed phase changes are contingent upon the initial phase configuration $(-3\pi /10\le \Psi \le 3\pi /10$) established at SLM (schematically shown in inset). **c** The ensuing alterations in the phase mapping of the speckle pattern within the designated areas (150 × 150 µm) highlighted in the $L{G}_{0}^{3}$ axial annular domain (see **a**), aligning with the prescribed initial phase configuration established at the SLM ($-3\pi /10\le \Psi \le 3\pi /10$). The upper and lower rows present, respectively, scenarios for low and multiple scattering environments; the scale bar corresponds to 150 µm. Video 1 presents the speckle pattern phase dynamics corresponding to the phase twist at the SLM

In a low scattering medium (*d*/*l*^∗^ ∼ 2), the LG beam propagates with minimal disruption, enabling it to maintain its initial OAM state and doughnut-like spatial intensity profile^36,41^, as well as the helical phase structure (Fig. 2a). The multiple scattering (*d*/*l*^∗^ ∼ 10) results in a diffusive spread of the LG beam’s intensity profile and the destruction of the helical phase front, leading to creation of complex speckle pattern (see Fig. 2a). Nevertheless, despite the influence of strong diffuse scattering, the phase speckle pattern maintains the modulation of the initial phase of the LG beam, representing a manifestation of the OAM’s memory in multiple scattering.

## Discussion

**Fig. 3:**
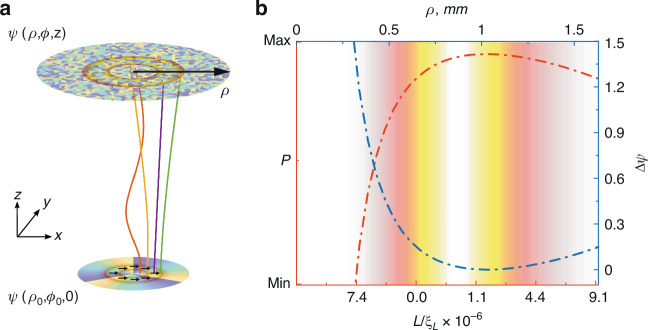
Polarization and phase evolution upon propagation of LG beam through a multiple scattering environment. **a** The spiral-like photon trajectories, schematically depicted in Cartesian coordinates, for the scalar linearly *x*-polarized $L{G}_{0}^{3}$ beam as if it were passing from the SLM to the axial annular zone (embossed by contours) at the detector through a medium devoid of scattering. **b** Radial distribution of the degree of polarization (*P*) and relative phase shift (ΔΨ) occurring along the open channel as propagated through a multiple scattering medium (*d*/*l*^∗^ ∼ 10) with the characteristic length of depolarization (*ξ*_*L*_). The colorful background represents the longitudinal profile of the intensity of the beam along the radial component (*ρ*)

The preservation of the helical phase structure by the LG beam along propagation through the scattering medium (see Fig. 2b, c) is attributed to the similarities in the deviations of the spiral-like photon trajectories around the beam axis caused due to rotational symmetry. Influenced by the scattering medium, the spiral-like photon trajectories significantly transform in relation to their original spiral paths (Fig. 3a), resulting in different partial components of the helical wavefront of the LG beam experiencing varying degrees of phase distortions. Due to the rotational symmetry, the deviations in these trajectories tend to be similar in magnitudes and directions around the beam axis. Therefore, despite the substantial distortions of phase induced by multiple scattering, the overall preservation of OAM of the LG beam is clearly observed in the phase dynamics of the speckle pattern, corresponding to the modulation of the initial phase at the SLM (see Fig. 2c and Video 1). Remarkably, during propagation through the multiple scattering medium (*d*/*l*^∗^ ∼ 10), the phase memory of OAM is distinctly pronounced within the axial annular region of the LG beam, as embossed in Fig. 2a (see also Video 1), with no discernible presence in the central and external areas beyond the annular region.

When light passes through a turbid tissue-like medium, even with a low scattering (*d*/*l*^∗^ < 2), the wavefront undergoes rapid deformation, resulting in a distinct speckle pattern. Notably, while traversing a more scattering medium, polarization scrambling occurs on a different length scale^42^, leading to the adoption of a chaotic or disordered nature in the polarization state of light. In other words, the initial orientation of the electric field vector representing the polarization of light undergoes unpredictable changes due to interactions with scattering elements in the medium. Polarization scrambling results in a loss of the initial alignment, leading to a more random distribution of polarization states. The degree of polarization of light is defined as the measure of the alignment of the electric field vectors within the light wave^43^:

5 $$\begin{array}{c}P=\frac{2L}{{l}_{s}}\sinh \left(\frac{{l}_{s}}{{\xi }_{L}}\right)\exp \left(-\frac{L}{{\xi }_{L}}\right)\end{array}$$

where *ξ*_*L*_ is the characteristic depolarization length $\left({\xi }_{L}={l}_{s}/\left(\sqrt{3\mathrm{ln}\frac{10}{7}}\right)\right)$, ${l}_{s}$ is the elastic mean free path $\left({l}_{s}={l}^{* }\left(1-{\rm{\langle }}\cos \theta {\rm{\rangle }}\right)\right)$, *θ* is the scattering angle, and *L* corresponds to the spiral-like photon trajectories (Eq. 3) of LG beam (see Fig. 3a).

In disordered scattering media, such as biological tissues and turbid tissuelike materials, light encounters multiple scattering events with various cellular components^44^, leading to the formation of branched transport channels^45^. In a similar fashion, the preservation mechanism within the axial annular region is associated with the exponential localization of the transmission eigenchannels, aligning with the shortest optical pathway^46^. Figure 3b shows a gradual transformation of polarization degree along the radial component (*ρ*) of the $L{G}_{0}^{3}$ beam, corresponding to the spread of the pathways ($L/{\xi }_{L}$) within the medium from SLM to the detecting area as presented in Fig. 3a. In other words, upon propagation of the scalar linearly *x*-polarized beam through multiple scattering medium (*d*/*l*^∗^ ∼ 10) the polarization remains effectively along the Eigenchannel, whereas the relative phase distortion $\Delta \Psi =\frac{\Psi {{\rm{|}}}_{L/{\xi }_{L}}-\Psi {{\rm{|}}}_{z/{\xi }_{L}}}{2\pi }$ becomes minimal within the axial annular zone (see Fig. 3b).

Eventually, upon propagation through a turbid tissue-like scattering medium (*d*/*l*^∗^ ∼ 10), the phase memory of OAM exhibits spatial variability when examined along *ρ*—the radial position (schematically shown in Fig. 3a). The overall helical phase structure associated with the OAM remains preserved, clearly observed in the single speckle grains within an arbitrarily chosen area within the annular region of the LG beam (see Fig. 2a). However, in the areas outside of the doughnut-like ring, the phase memory effect disappears. Conversely, in a low-scattering environment (*d*/*l*^∗^ ∼ 2), the phase memory is more consistently observed in all regions (see Fig. S2 in Supplementary Note 2 and Video 2). Computational analyses corroborate these experimental findings, affirming that the phase memory of OAM in LG beams propagated through turbid tissue-like scattering media is not absolute. It is susceptible to various factors, including scattering, absorption, and anisotropy of scattering, potentially leading to the degradation or alteration of the OAM’s phase content.

Thus, in the current exploration of the phase preservation of OAM light within multiple scattering environments, we draw upon established biophotonics principles, particularly the dimensionless optical depth (*d*/*l*^∗^) guideline. This guideline categorizes scattering regimes into single, intermediate (‘snake-like’), and multiple or diffuse scattering based on the ratio of the medium’s thickness to the transport mean free path of photons. Our findings bridge a crucial gap for the biophotonics and OAM communities by demonstrating how OAM light, even in environments characterized by a high optical depth (*d*/*l*^∗^ ≈ 10), retains its helical phase structure—a phenomenon directly linked to the rotational symmetry and uniform deviations in the spiral-like photon trajectories around the beam axis (as schematically illustrated in Video 3). This behavior underscores the potential of OAM light for probing biological tissues in the multiple scattering regime, where conventional optical methods are challenged by extensive photon path randomization and phase information loss. The preservation of topological integrity by OAM light across diverse scattering scenarios enhances understanding of the interactions between light and complex media, opening new options for innovative advancements in optical sensing and imaging in the biomedical domain.

Leveraging the unique interaction mechanisms of OAM light within tissuelike scattering media presents an unparalleled opportunity to discern changes in the refractive index of cellular environment^47^, arguably cellular cytoplasm and extracellular matrix. This novel integration of OAM within scattering environments notably amplifies our capacity to investigate the interactions of quasi-ballistic photons with a diverse array of cellular components. This methodology not only permits a meticulous analysis of variations in the refractive index of cells, potentially indicative of underlying biological or pathological transformations but also enhances the interaction dynamics of quasi-ballistic photons through OAM, facilitating a non-intrusive technique to evaluate the inherent optical properties of cellular constituents. Such a technique, with its non-invasive nature and enhanced sensitivity, is set to revolutionize our understanding of cellular modifications, thereby opening new avenues for comprehending health and disease mechanisms at the cellular echelon.

While our study underscores the potential of exploiting OAM light for probing and diagnostic applications, it is imperative to acknowledge the inherent limitations posed by scattering, absorption, and anisotropy of scattering. For instance, our observations revealed that in low-scattering environments (*d*/*l*^∗^ ≈ 2), the LG beams retained their helical phase structure, whereas in multiple scattering conditions (*d*/*l*^∗^ ≈ 10), the phase content experienced degradation, leading to complex speckle patterns (refer to Fig. 2a). This exemplifies the sensitivity of OAM light to the scattering properties of the medium, affecting its phase preservation capabilities. Anisotropy of scattering further complicates this interaction, as it influences the directional spread of light within the medium^48,49^, altering the phase content of OAM light. These factors collectively highlight the challenges in maintaining the integrity of OAM’s phase structure in optically complex media, marking a critical area for future research to optimize OAM-based technologies for real-world applications.

In the current study, we introduce a pioneering approach to probing complex media by harnessing the distinctive properties of OAM light. Unlike traditional phase patterns, which primarily offer phase change detection capabilities, OAM beams are endowed with helical phase fronts that exhibit unparalleled sensitivity to the minutest refractive index variations within the medium. This sensitivity is crucial for applications demanding high precision, such as non-invasive medical diagnostics, where detecting subtle changes in biological tissues can provide critical insights into physiological conditions or disease states.

Moreover, the choice of OAM light extends beyond its sensitivity to its inherent topological robustness. OAM beams maintain their encoded information—the helical phase structure—despite encountering complex scattering phenomena that typically distort or degrade other types of light beams. This topological preservation of phase information, a property unique to OAM light, ensures that the probing signal retains its integrity, thereby enhancing the reliability and accuracy of measurements in turbid or optically dense environments. This characteristic is not merely advantageous but is paramount in environments where traditional light beams would fail to provide consistent and interpretable data.

Our work leverages these unique aspects of OAM to develop a novel probing methodology that significantly advances the capability to investigate complex media with unprecedented detail and sensitivity. Through experimental and theoretical analyses, we demonstrate the superior performance of OAM-based probing in detecting and analyzing variations within scattering media, setting a new precedent for optical probing technologies. This novel application of OAM in probing underlines a significant shift from its traditional roles in optical communications and manipulation, showcasing its versatility and untapped potential in scientific exploration and diagnostics.

The study underscores the crucial role of topological preservation in OAM light for probing complex media. Unlike conventional light sources, OAM light maintains its unique helical phase structure and topological charge through scattering environments, enabling unparalleled precision in detecting subtle changes within the medium. By leveraging the robustness of OAM light’s topological features, our research introduces a novel approach to optical probing, offering deeper insights into the microscopic properties of complex systems.

The obtained results offer fascinating opportunities for exploiting OAM light in biomedical applications, e.g., such as non-invasive trans-cutaneous glucose diagnosis and optical communication through biological tissues and other dispersed multiple-scattering materials. The non-invasive nature of OAM light probing is especially beneficial for continuous monitoring and diagnostics in vulnerable populations, including pediatric, elderly, or immunocompromised patients, where traditional invasive procedures are less feasible. Additionally, the ability to structure and tune OAM light in complex ways not only enhances its application in diagnostic imaging, such as in optical coherence tomography (OCT) and microscopy^12^, but also extends its utility to therapeutic uses, including targeted photodynamic therapy^50^. Utilizing OAM light in quantitative phase imaging (QPI)^51^ further exemplifies its value in live cell imaging and dynamic biological process monitoring, providing essential non-invasive insights into cell health, function, and structure. Additionally, the intrinsic sensitivity of OAM beams to refractive index changes makes them highly effective in detecting subtle physiological alterations that could indicate the early stages of diseases such as cancer or diabetes.

By manipulating the wavefront shaping of OAM beams, it’s possible to customize penetration depth and focal precision within tissues^52^, thereby facilitating selective imaging at varying depths without the necessity of physically adjusting the imaging equipment. This capability allows for detailed examination of layered tissues like skin or the retina, enhancing diagnostic accuracy and patient outcomes. Furthermore, the modulation of OAM modes can alter the light-tissue interaction dynamics, significantly improving the contrast in images, and aiding in distinguishing between normal and pathological tissue structures.

Given these properties, our study underscores the profound implications of employing OAM light in medical diagnostics, providing a pathway to safer, more accurate, and earlier detection of pathological changes within the body. This approach holds promise not only for enhancing existing diagnostic techniques but also for pioneering novel applications in areas such as single molecule imaging and the study of single-cell dynamics, which are critical for advancing our understanding of cellular processes and disease progression.

Moreover, the OAM-based diagnostic approach detailed in our research, particularly through the analysis of blood smears^53^, demonstrates a novel method to detect early signs of diseases such as cancer by exploiting the unique optical anisotropy properties of blood proteins. These capabilities make OAM an invaluable tool in the ongoing development of more effective medical diagnostic technologies.

## Materials and methods

### Optical setup and processing

**Fig. 4:**
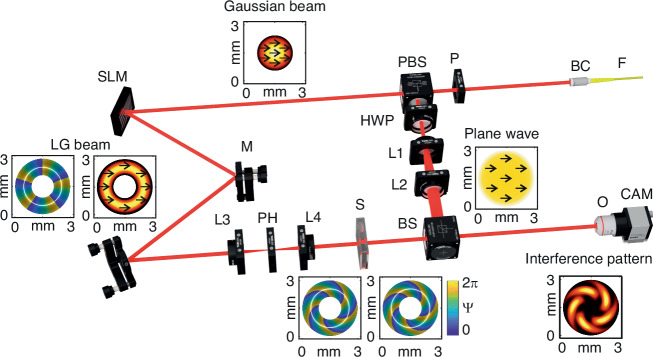
Experimental setup. LD laser diode, P polarizer, FM fiber mount, BC beam collimator, SLM spatial light modulator, M1, M2 mirrors, L1, L2, L3, L4 lenses, PH pinhole, PBS polarizing beam splitter, HWP half-wave plate, S cuvette filled with the sample liquid, BS beam splitter, NF neutral filter, O objective, and CCD camera. A detailed description of the optical setup is presented in the main article text

The experimental configuration employed for measurements is schematically illustrated in Fig. 4. Here, the modified Mach–Zehnder-based interferometer^54^ is used to examine the evolution of OAM of the LG beams propagated through the multiple scattering environments (Fig. 4).

A coherent Gaussian light beam (40 mW, BioRay laser diode, Coherent, USA with coherence length *>* 20 cm) emitting at 640 nm serves as the light source. To clear the optical mode, the laser beam is focused into a single-mode optical fiber (P1-630A-FC-1, Thorlabs, USA) F. The resulting output beam is collimated using a beam collimator (F280FC-B, Thorlabs, USA) BC to obtain a Gaussian beam with a 1.6 mm waist diameter. Additionally, a polarizer (Thorlabs, USA) P is used after the collimator to achieve horizontal linear polarization. The Gaussian beam is then split into sample and reference beams using a polarizing beam splitter (Thorlabs, USA) PBS. A SLM (PLUTO-2-NIR-011, Holoeye, Germany) is utilized in the reference arm to generate various LG beams carrying OAM. These OAM-carrying beams traverse the sample—scattering environment. Simultaneously, the reference Gaussian beam undergoes expansion through a series of lenses and is directed to a beam splitter, where it interferes with the sample beam. Subsequently, the interference pattern is recorded using a charge-coupled device (CCD) camera. The sample beam illuminates the phase-only SLM (PLUTO-2-NIR-011, Holoeye, Germany) SLM, operating in a reflective regime. To produce LG beams with different moments, the corresponding forked diffraction patterns are generated on the SLM. The diffracted light from the SLM is directed using a set of mirrors M to lens L3 (*f* = 45 mm, Thorlabs, USA). This lens is used to focus the first-order diffraction through a pinhole (Thorlabs, USA). The LG beam is then re-collimated using lens L4 (*f* = 45 mm, Thorlabs, USA). Finally, the sample beam passes through sample S. The reference beam goes through a half-wave plate (Thorlabs, USA) to control the polarization orientation of the Gaussian beam. This reference beam is expanded by lenses L1 (*f* = 30 mm Thorlabs, USA) and L2 (*f* = 70 mm, Thorlabs, USA), and directed to the beam splitter (Thorlabs, USA) BS, where the expanded Gaussian beam interferes with the LG beam. The interference pattern is then registered using a CMOS camera (DCC3240M, 1280 × 1024, Thorlabs, USA) CAM in combination with an objective (10×, Nikon, Japan) O. The waist beam diameter for $L{G}_{0}^{3}$ and $L{G}_{0}^{5}$ beams at the detector is 2.7 mm and 3 mm, respectively.

The setup enables the acquisition of interference patterns in both on-axis^55^ and off-axis^56^ regimes. In the on-axis scenario, the definition of relative OAM twist involves tracking the change in the polar angle of a petal caused by its rotation around the center of the LG beam. Meanwhile, in the off-axis regime, the retrieval of the LG beam phase is accomplished through a fast Fourier transform approach^56^.

### Samples: scattering environment

The selection of the ethanol–water solution as an optically transparent medium for experimental inquiry is underpinned by careful consideration. The quantification of the relative OAM twist, stemming from temperature-dependent changes in the refractive index of the solution, is systematically conducted. A precisely measured volume of 5 ml of an ethanol–water solution, characterized by a water concentration of 51.89 mol%, is carefully introduced into a glass cuvette. The cuvette, with a total thickness of 5.6 mm, features glass walls, each having a thickness of 1 mm.

The investigation thoroughly addresses the intricate thermal dependencies governing the refractive index of the ethanol–water amalgamation, as comprehensively detailed in the authoritative work^57^. Subsequently, the cuvette containing the liquid sample undergoes refrigeration until reaching a temperature of $7.8\pm {0.5}\,^{\circ }{\rm{C}}$. After the cooling phase, the cuvette is reintegrated into the experimental apparatus and subjected to a 25-min duration at room temperature (${21}\,^{\circ }{\rm{C}}$). This deliberate protocol induces modulations in the refractive index of the sample liquid during the heating process.

The nuanced alterations in interference patterns, reflective of variations in the refractive index, are meticulously captured by a high-speed camera throughout the entirety of the 25-min heating interval. The recording is conducted at a frame rate of ten frames per second, each frame having a 1 ms exposure time. These experimental procedures adhere to rigorous standards to ensure the fidelity and reliability of the acquired data.

Throughout the experimental duration, the sample temperature is vigilantly monitored at 10-s intervals utilizing a FLIR C5 thermal camera from Teledyne FLIR, USA.

The measurements are conducted in a low scattering (*d*/*l*^∗^ = 2) phantom with a thickness of 1 mm (*µ*_*s*_ = 10 mm^−1^) and the multiple-scattering one (*d*/*l*^∗^ ∼ 10) with a thickness of 8 mm (*µ*_*s*_ = 6 mm^−1^). The meticulous methodology employed in the preparation of the phantoms is comprehensively expounded upon in the work by Wrobel et al., as documented in ref. ^58^. For the experimental measurements involving biological tissues, the chicken skin was utilized. These samples were sourced from a local supplier and delivered to our laboratory under controlled conditions to ensure freshness and consistency. The experiments were conducted on the same day of delivery to preserve the biological integrity of the samples, allowing for an accurate representation of skin tissue properties in our study.

### Computational modeling

Due to the complexity of tissue-like turbid media, which feature strong light scattering and high anisotropy, traditional analytical methods for describing shaped light propagation are largely ineffective. Consequently, stochastic methods like Monte Carlo (MC) simulations have become the preferred tools for modeling light propagation in such environments, serving as the ‘gold standard’ for investigating photon transport in biological tissues^59^.

In our studies, we employed a semi-analytical MC approach^60^. This method calculates the likelihood of photon propagation directly to a detector from each scattering point before the photon packet enters the detector’s defined region, shaped by its physical dimensions and numerical aperture. This semi-analytical approach has been validated in various studies, including signal formation in OCT^61^, laser beam propagation through scattering media^62,63^, and modeling laser speckle patterns^64^. It combines the exact analytic Milne solution with the iterative Bethe–Salpeter equation^37,65,66^ and Jones vector formalism, effectively tracking the polarization of MC-photons within turbid media and simulating coherent backscattering^67,68^. This approach has also been adapted to include polarized light, expanding its use in biomedical optics diagnostics^35^. The foundation for these polarized MC approaches is laid by the vector radiative transfer equation, derived from Maxwell’s electromagnetic theory^69–71^. Including shaped light-carrying OAM in MC modeling^36,72,73^ is gaining recognition for its potential to enhance diagnostic tools by providing deeper insights into the structural complexities of turbid tissue-like media and addressing current limitations.

In our study, the computational analysis that supports and validates our experimental findings is based on a hybrid approach that integrates a semianalytical inversion scheme to calculate the modulation transfer function of a turbid medium^74^ with vector-based MC^35–37^. This approach simulates the propagation of shaped polarized light as it interacts with scattering and absorbing media, ideal for modeling the intricate dynamics of LG beams endowed with OAM. By tracing the trajectories of individual photons and considering the effects of scattering, absorption, their mutual interference, and the helical phase structure of the beams, our computational model provides a detailed understanding of how OAM features are preserved in various media.

Expressions (Eqs. 1 and 2) describe the intensity and phase distributions of the LG beam in the medium with *n* = 1. To assess the conversion of OAM in the tissue-like scattering medium (*n* / = 1) a large set (e.g., *N*_*ph*_ ∼ 10^9^) of LG beam photon trajectories with starting points ${\rho }_{{0}_{i}},{\phi }_{{0}_{i}},i\in \left[1...{N}_{{ph}}\right]$ is generated^36^. Azimuths phase for each trajectory can be easily calculated as −*ℓϕ* as follows from (Eq. 2) in the main text. The length *L*_*i*_ of each trajectory is estimated according to (Eq. 3). Within the cuvette (or within the sample) we estimate trajectory lengths as $\Delta {L}_{i}\left({r}_{{0}_{i}},{\varphi }_{{0}_{i}}\right)=L\left({r}_{{0}_{i}},{\varphi }_{{0}_{i}},{\zeta }_{2}\right)-L\left({r}_{{0}_{i}},{\varphi }_{{0}_{i}},{\zeta }_{1}\right)$, where ${\zeta }_{2}-{\zeta }_{1}$ corresponds to cuvette (sample) thickness in dimensionless units introduced in (Eq. 3) (see the main text). Due to the non-zero contrast between refractive indices of free space, glass, and interior of the cuvette (sample), the trajectories are additionally refracted according to Snell’s law, slightly increasing $\Delta {L}_{i}$ values.

We estimate cuvette (sample) influence on the LG beam propagation via phase retardation caused by the increasing path length of light within the medium:

6 $$\Delta {\Psi }_{i}=\frac{2\pi n\Delta {L}_{i}}{\lambda }$$

Here, $\Delta {\Psi }_{i}$ corresponds to the phase retardation along *i*’th trajectory. By comparing the phase before cuvette $\varPsi {{\rm{|}}}_{{L}_{i}}$ and phase after cuvette $\Psi {{\rm{|}}}_{{L}_{i}+\varDelta {L}_{i}}=\Psi {{\rm{|}}}_{{L}_{i}}+\Delta {\Psi }_{i}$ for different values of *n*, we obtain phase patterns that are twisted differently, as seen in Fig. 1 in the main text.

Upon computation of the intensity and phase beyond the cuvette (sample), it becomes feasible to derive the interference pattern of LG beams with a plane wave, yielding the characteristic petal pattern on the screen with |*ℓ*| petals. These petals undergo a twist in correspondence to variations in the refractive index *n* within the cuvette interior. The quantification of this twist is elucidated in the ensuing procedure, and the obtained results exhibit notable concordance with experimental measurements, as illustrated in Fig. 1 in the main text.

The proposed computational methodology facilitates the assessment of pathlengths $\Delta {L}_{i}$ in scenarios where the cuvette (sample) interior manifests turbidity. In such instances, the trajectories of LG beams are construed in the context of MC photons^36^, undergoing multiple scattering events dictated by the medium’s scattering coefficient *µ*_*s*_, absorption coefficient *µ*_*a*_, and anisotropy of scattering parameter *g* (*g* = 〈cos*θ*〉).

### Characterization of twist of the LG beam propagated through the medium

The quantification of the relative OAM twist in the on-axis regime is established by defining the change in the polar angle of a petal due to its rotation around the center of the LG beam. To ascertain this twist, a polar coordinate system is introduced, with the origin situated at the center of the LG beam for each recorded experimental image. Subsequently, a binary representation is generated for each image by binarizing the intensity values, with those exceeding a defined threshold set to one, and all other values set to zero. The luminance threshold is determined utilizing the Otsu approach^75^, and can be manually adjusted either for an individual image or across a series of images to ensure clarity in visualizing each petal on the binary representation.

The identification of pixels corresponding to the outer boundaries of each illuminated region in the binary image is achieved through the utilization of the Moore-Neighbor tracing algorithm, adapted to adhere to Jacob’s stopping criteria^76^. The preeminent bright spots are associated with the interference pattern petals of the LG beam, contingent upon the selection of an optimal contrast threshold. All other bright spots are deemed artifacts, and their respective boundary pixels are systematically excluded from subsequent data analysis.

**Fig. 5:**
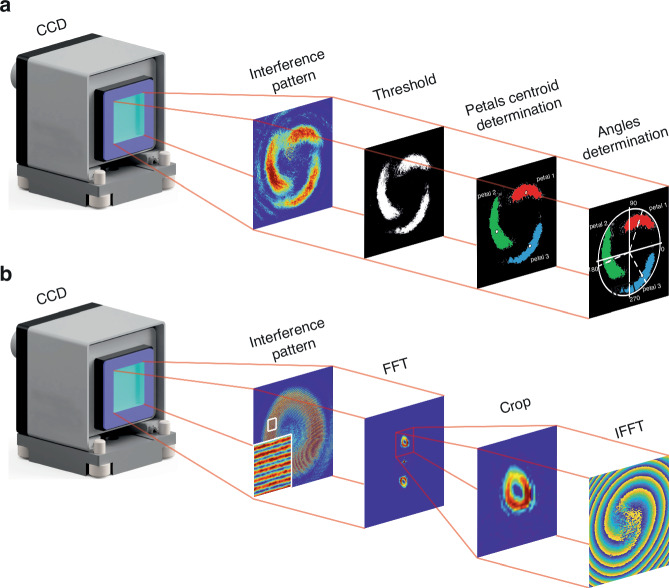
Analysis of the LG beam interference patterns. Schematic presentation of **a** main steps and principles for determining the relative twist of petals in the on-axis regime; **b** main steps and principles for the phase retrieval procedure in the off-axis regime

By discerning the boundary pixels, individual polygons are meticulously constructed for each petal, preserving their inherent geometric characteristics. For every polygon, the polar coordinates of its centroid are calculated. The derivation of final relative twist values is accomplished by subtracting the angular coordinates of the centroids between different images, considering the spatial orientation of the petals, and ensuring a seamless 0-to-2*π* transition. A schematic representation delineating the underlying principles and key steps of this method is presented in Fig. 5a.

### Phase retrieval

In the off-axis regime, the retrieval of the LG beam phase is accomplished through a straightforward signal processing approach, as elucidated in the work by Vayalamkuzhi et al. ^56^. The schematic illustrating the principal steps of this procedure is presented in Fig. 5b. Initially, a fast Fourier transform is employed on the detected interference pattern with the primary objective of extracting the frequency spectrum. Subsequently, a judicious selection is made to isolate the pertinent frequency spectrum corresponding to the LG beam, demarcated by the red square in the FFT image.

The utilized setup facilitates the acquisition of interference patterns in both on-axis^55^ and off-axis^56^ regimes. The determination of relative OAM twist in the on-axis regime involves assessing the change in the polar angle of a petal caused by its rotation around the LG beam center. Conversely, in the off-axis regime, the LG beam phase is retrieved through a fast Fourier transform approach^56^. A detailed elucidation of the experimental data processing methodology is presented above.

### Supplementary information

Supplementary

Video 1

Video 2

Video 3

## Data Availability

All data related to the experiments and computational modeling described in this article are archived on a lab computer at the University of Oulu. All data are available from the corresponding author upon reasonable request.

## Code availability

The codes used for the modeling of conversion of OAM for different types of LG beams and evaluation of the twist of OAM in experimental studies are available from the corresponding author upon reasonable request.
