# Supplementary material for: Phase preservation of orbital angular momentum of light in multiple scattering environment
Source: Light Sci Appl. 2024 Aug 26;13:214. doi: 10.1038/s41377-024-01562-7 (PMC11347564; doi:10.1038/s41377-024-01562-7)
Supplement: Supplementary file 1 — Supplementary [file 41377_2024_1562_MOESM1_ESM.docx]

# Supplementary information for

# Phase Preservation of Orbital Angular Momentum of Light in Multiple Scattering Environment

Igor Meglinski^1*^, Ivan Lopushenko^2^, Anton Sdobnov^2^ and Alexander Bykov^2*^

^1^College of Engineering and Physical Sciences, Aston University, Birmingham, B4 7ET, UK.

^2^Optoelectronics and Measurement Techniques, University of Oulu,
P.O. Box 4500, Oulu, FI-90014, Finland.

*Corresponding authors. E-mails:

i.meglinski@aston.ac.uk; alexander.bykov@oulu.fi.

Contributing authors. E-mails:
ivan.lopushenko@oulu.fi; anton.sdobnov@oulu.fi.

This PDF file includes:

Supplementary Figures S1, S2 and S3

**Supplementary Text**

**Supplementary Note 1: Addition to Figure 2**

**Fig. S1**: **Composition of LG beam’s helical structure evolves upon propagation through the medium**


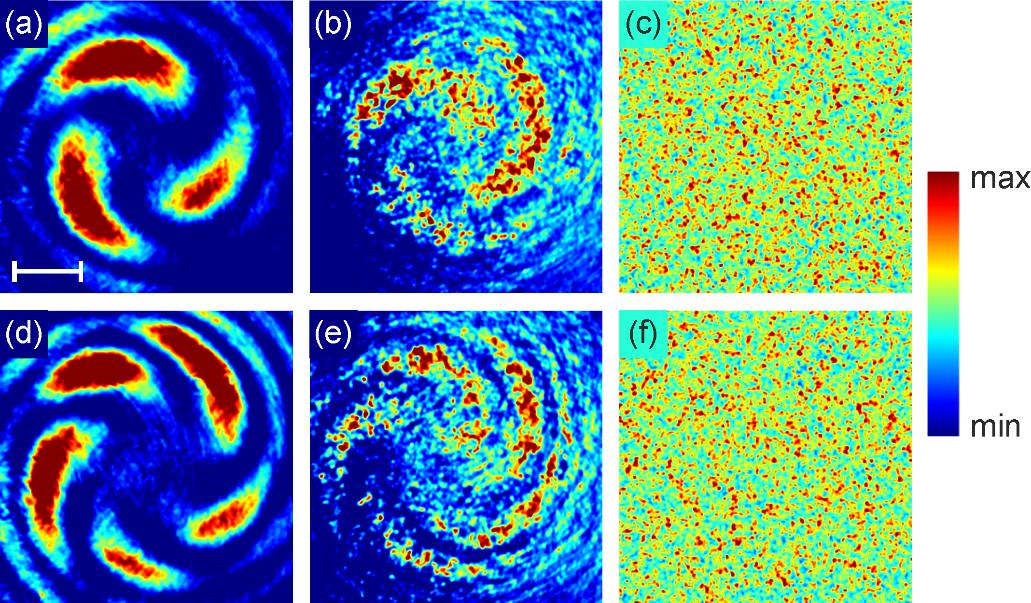


Experimentally observed spatial intensity distributions for $LG_{0}^{3}$ and $LG_{0}^{5}$ beams propagated through a transparent medium ((**a**) and (**d**)), as well as through the low (*z/l*^∗^ = 2) and multiple (*z/l*^∗^ = 9*.*6) scattering environments, respectively, ((**b**) and (**e**)) and ((**c**) and (**f**)). Scale bar is equal to 750 µm.

**Supplementary Note 2: Addition to Video 2**

**Fig. S2**: **The phase memory of OAM in scattering environment**


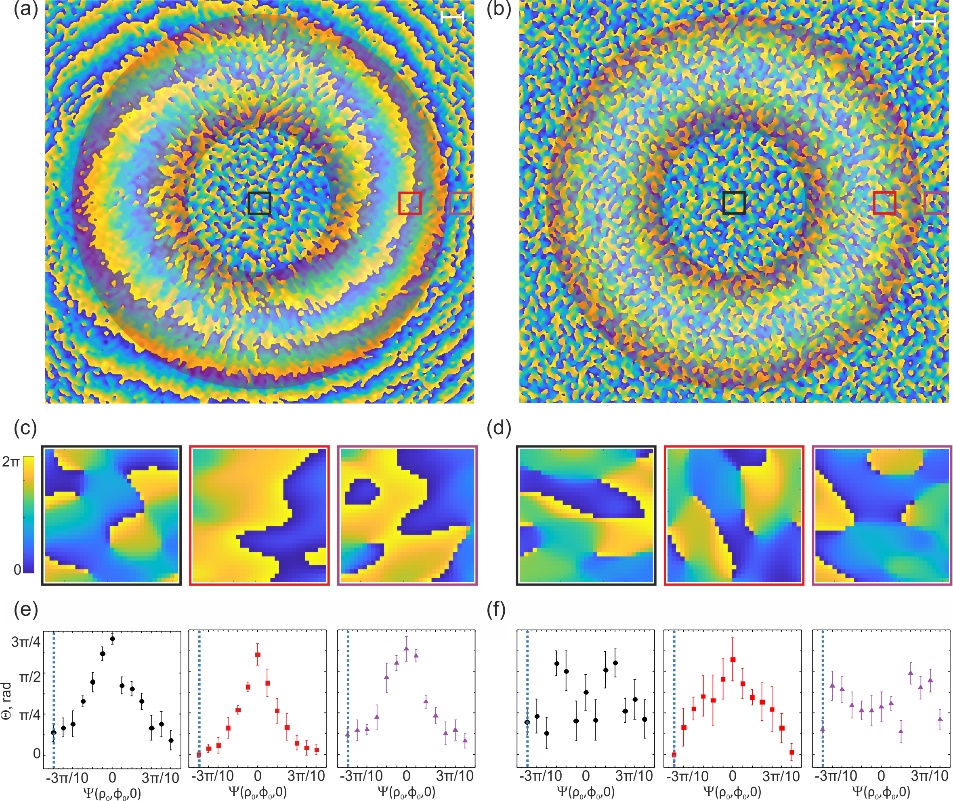


Phase distribution (speckle patterns) observed experimentally for the $LG_{0}^{3}$ beam propagated through the low (*d/l*^∗^ = 2) scattering (**a**) and multiple
(*d/l*^∗^ = 9*.*6) scattering (**b**) media. The axial annular zone (embossed by contours) corresponds to the $LG_{0}^{3}$ beam as if it were passing through a medium devoid of scattering. The resulting changes in the phase distribution of speckle patterns observed within specified areas (size: 150×150 µm) selected at the center (left), axial annular region (center), and outside (right) of the $LG_{0}^{3}$ beam profile (as indicated in (**a**) and (**b**)), presented for both low scattering (**c**) and multiple scattering (**d**) environments. Respectively, the phase variations observed at the single speckle grain within center (left), axial annular region (center), and
outside (right) of the $LG_{0}^{3}$ beam both low (**e**) and multiple (**f**) scattering. The observed phase changes are contingent upon the initial phase configuration (−3*π/*10 ≤ Ψ ≤ 3*π/*10) established at SLM see Video 2.

**Supplementary Note 3: Addition 2 to Figure 2**

**Fig. S3**: **The phase memory of OAM in various scattering environment**


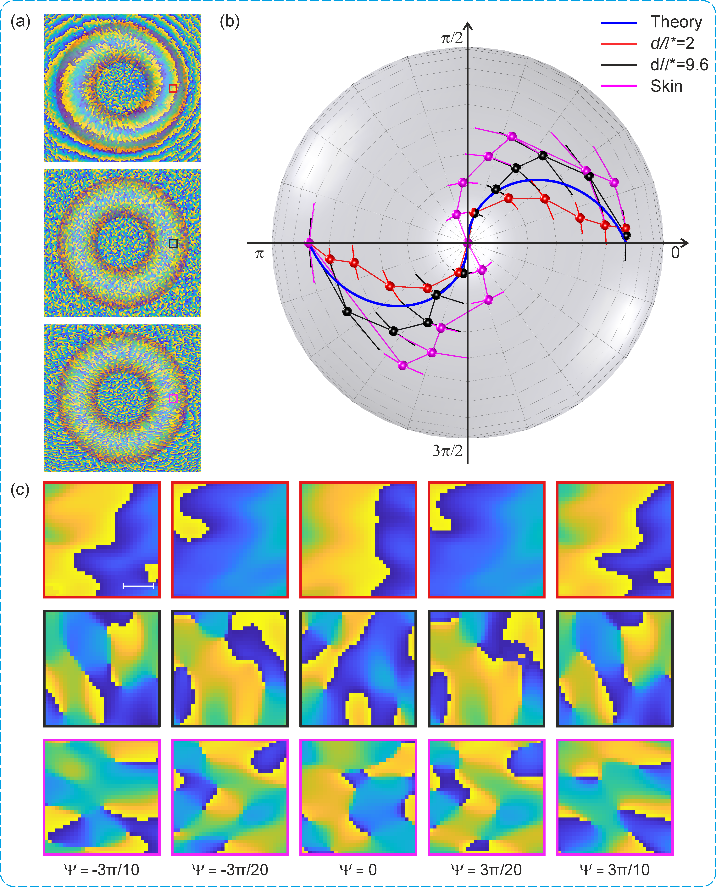


**a**, Phase distribution (speckle patterns) observed experimentally for the $LG_{0}^{3}$ beam propagated through phantoms with the low (*d/l*^∗^ = 2) scattering (top), multiple (*d/l*^∗^ = 9*.*6) scattering (middle) and biological tissue – chicken skin (bottom). The axial annular zone (embossed by contours) corresponds to the $LG_{0}^{3}$ beam as if it were passing through a medium devoid of scattering. **b**, Phase variations manifest at the single speckle grain within a deliberately chosen sector of the $LG_{0}^{3}$ axial annular area for low-scattering (indicated by black circles) and multiple-scattering (represented by red circles) media and skin (magenta circles). The observed phase changes are contingent upon the initial phase configuration (−3*π/*10 ≤ Ψ ≤ 3*π/*10) established at SLM (schematically shown in inset). **c**, the ensuing alterations in the phase mapping of the speckle pattern within the designated areas (150 × 150 µm) highlighted in the $LG_{0}^{3}$ axial annular domain (see Fig. 2-a), aligning with the prescribed initial phase configuration established at the SLM (−3*π/*10 ≤ Ψ ≤ 3*π/*10). The upper, middle and lower rows present, respectively, scenarios for phantoms with low, multiple scattering and skin; scale bar corresponds to 150 µm.
